# Genome Sequence of the ε-Poly-l-Lysine-Producing Strain *Streptomyces albulus* NK660, Isolated from Soil in Gutian, Fujian Province, China

**DOI:** 10.1128/genomeA.00532-14

**Published:** 2014-06-12

**Authors:** Yanyan Gu, Chao Yang, Xiaomeng Wang, Weitao Geng, Yang Sun, Jun Feng, Yuanyuan Wang, Yufen Quan, You Che, Chi Zhang, Ting Gong, Wei Zhang, Weixia Gao, Zhenqiang Zuo, Cunjiang Song, Shufang Wang

**Affiliations:** a Key Laboratory of Molecular Microbiology and Technology for Ministry of Education, Nankai University, Tianjin, China; b State Key Laboratory of Medicinal Chemical Biology, Nankai University, Tianjin, China

## Abstract

We determined the complete genome sequence of a soil bacterium, *Streptomyces albulus* NK660. It can produce ε-poly-l-lysine, which has antimicrobial activity against a spectrum of microorganisms. The genome of *S. albulus* NK660 contains a 9,360,281-bp linear chromosome and a 12,120-bp linear plasmid.

## GENOME ANNOUNCEMENT

*Streptomyces* spp. are Gram-positive, soil-dwelling, filamentous bacteria, which are responsible for producing a wide variety of economically important natural secondary metabolites used in human and veterinary medicine (1). ε-Poly-l-lysine (ε-PL), which consists of 21 to 35 l-lysine residues with linkages between the α-carboxyl groups and ε-amino groups, is produced by bacteria belonging to the *Streptomycetaceae* family (2). ε-PL exhibits antimicrobial activity against a wide spectrum of microorganisms, including Gram-positive and Gram-negative bacteria; it also exhibits antiphage activity. In addition, due to its safety and biodegradability, ε-PL has been used as a food preservative in several countries, such as Japan, South Korea, and the United States (3, 4).

We report here the genome sequence of the ε-PL-producing strain *Streptomyces albulus* NK660, which was isolated from Gutian, Fujian Province, China. The structure of ε-PL was determined by matrix-assisted laser desorption ionization–time of flight mass spectrometry (MALDI-TOF MS) and gel permeation chromatography (GPC). These results showed that the polymerization degree of the ε-PL produced by strain NK660 ranges from 19 to 33 l-lysine monomers, and its molecular mass range is between 2,453 and 4,248 Da (5). The strain is currently maintained at the China General Microbiological Culture Collection Center (CGMCC no. 5392).

The genomic sequence of *S. albulus* NK660 was obtained by the assembly of three data sets, which were generated by the Roche 454 GS-FLX system, a Solexa analyzer (Illumina), and single-molecule real-time sequencing (PacBio). All of the paired reads were assembled using the SMRT Portal version 2.2.0. Annotation was done by merging the results obtained from Prodigal (6), RepeatMasker, tRNAscan-SE 1.21 (7), and RNAmmer 1.2 (8). Gene functional annotation was based on BLASTp with the Nonredundant (NR) databases, Kyoto Encyclopedia of Genes and Genomes (KEGG) (9), Protein Families (PFAM) database (10), and Clusters of Orthologous Groups (COG) (11).

The genome of *S. albulus* NK660, with a G+C content of 72.32%, comprises a 9,360,281-bp linear chromosome and a 12,120-bp linear plasmid. The chromosome contains 7 rRNA operons, 70 tRNA genes, and 8,190 protein-coding genes (CDSs), holding about 85.32% of the whole genome. Among the 8,190 open reading frames (ORFs), 4,978 (60.78%) have a clear function, 573 (7.00%) encode products with high similarity to putative proteins, and the remaining 2,639 (32.22%) have no match in the protein database of the National Center for Biotechnology Information (NCBI).

Yamanaka et al. (12) clarified the catalytic mechanisms of ε-PL synthetase (Pls). Pls is a membrane protein with six transmembrane domains surrounding three tandem soluble domains. These tandem domains iteratively catalyzed l-lysine polymerization using free l-lysine polymer as the receptor and T-domain-bound l-lysine as the donor, directly yielding chains of different lengths. However, additional details on the regulation of ε-PL biosynthesis need further investigation. Therefore, this draft genome sequence will allow for developing a deeper understanding of its regulatory mechanism, thereby allowing us to construct an ε-PL overproducer for large-scale industrial production.

### Nucleotide sequence accession numbers.

The genome sequence of *S. albulus* NK660 has been deposited in GenBank, where the linear chromosome accession no. is CP007574 and the linear plasmid sequence accession no. is CP007575.
